# Scoping review of telehealth use by Indigenous populations from Australia, Canada, New Zealand, and the United States

**DOI:** 10.1177/1357633X231158835

**Published:** 2023-03-13

**Authors:** Débora Petry Moecke, Travis Holyk, Madelaine Beckett, Sunaina Chopra, Polina Petlitsyna, Mirha Girt, Ashley Kirkham, Ivan Kamurasi, Justin Turner, Donovan Sneddon, Madeline Friesen, Ian McDonald, Nathan Denson-Camp, Stephanie Crosbie, Pat G Camp

**Affiliations:** 1 University of British Columbia (UBC), Vancouver, Canada; 2 Faculty of Medicine, 8166 University of British Columbia, Vancouver, Canada; 3 Carrier Sekani Family Services, Prince George, Canada; 4 1974 Faculty of Medicine, University of Queensland, Brisbane, Australia

**Keywords:** Virtual care, cultural safety, cultural appropriateness, therapeutic relationships, Indigenous health, telehealth

## Abstract

**Introduction:**

Telehealth has the potential to address health disparities experienced by Indigenous people, especially in remote areas. This scoping review aims to map and characterize the existing evidence on telehealth use by Indigenous people and explore the key concepts for effective use, cultural safety, and building therapeutic relationships.

**Methods:**

A search for published and gray literature, written in English, and published between 2000 and 2022 was completed in 17 electronic databases. Two reviewers independently screened retrieved records for eligibility. For included articles, data were extracted, categorized, and analyzed. Synthesis of findings was performed narratively.

**Results:**

A total of 321 studies were included. The most popular type of telehealth used was mHealth (44%), and the most common health focuses of the telehealth interventions were mental health (26%) and diabetes/diabetic retinopathy (13%). Frequently described barriers to effective telehealth use included concerns about privacy/confidentiality and limited internet availability; meanwhile, telehealth-usage facilitators included cultural relevance and community engagement. Although working in collaboration with Indigenous communities was the most frequently reported way to achieve cultural safety, 40% of the studies did not report Indigenous involvement. Finally, difficulty to establish trusting therapeutic relationships was a major concern raised about telehealth, and evidence suggests that having the first visit-in-person is a potential way to address this issue.

**Conclusion:**

This comprehensive review identified critical factors to guide the development of culturally-informed telehealth services to meet the needs of Indigenous people and to achieve equitable access and positive health outcomes.

## Introduction

Equitable access to health services is a fundamental human right.^1^ To address health disparities and improve accessibility to healthcare services, several initiatives have been adopted by many countries,^2–5^ including telehealth. Telehealth is a treatment delivery mechanism that involves the remote exchange of data between patients and healthcare providers and has reduced barriers to accessing healthcare services faced by diverse equity-seeking populations around the world,^6^ including Indigenous populations.^7–9^ However, to be effective, telehealth needs to be tailored to the specific social and cultural contexts of the Indigenous communities to which it serves.^10^ Various aspects of Indigenous culture (i.e., identity, spirituality, traditional healing practices) are known as sources of strength and resilience for Indigenous peoples, acting as protective mechanisms to buffer the negative influences of historical trauma on Indigenous health outcomes.^11,12^ However, western biomedical health care models may not value culture as a component necessary for the successful delivery of telehealth care services. Indeed, in various parts of the world Indigenous peoples’ cultural characteristics and practices were targeted by historical colonial persecution^13–15^ and continue to be the object of ongoing discrimination and racism.^16,17^ Therefore, considering the role of culture is a key aspect when exploring strategies to overcome barriers that Indigenous people face when accessing health services.

Despite the growing body of literature supporting the use of telehealth for Indigenous populations, there are still knowledge gaps. Previous reviews have focused on specific diseases, health conditions, and types of technologies,^18–21^ but have not broadly characterized the use of telehealth for Indigenous populations or explored cultural safety and therapeutic relationship aspects. This scoping review will map and characterize the existing knowledge on telehealth for Indigenous peoples in Australia, Canada, New Zealand, and the US, and explore the key concepts for effective use, cultural safety, and building therapeutic relationships.

## Methods

The study protocol for this scoping review has been previously published.^22^ This review was conducted in accordance with the Joanna Briggs Institute Reviewers’ Manual^23^ and supplemented by the methodological frameworks developed by Arksey and O’Malley^24^ and further refined by Levac et al.^25^ The research question was developed using a PCC (Population, Concept, Context) mnemonic: What are the characteristics of telehealth (concept) use by Indigenous adult populations (population) in Australia, Canada, New Zealand, and the US (context)? Data will be reported using PRISMA extension for scoping reviews (PRISMA-ScR).^26^ We selected these four countries due to their similar histories of colonialism by English European powers and present-day statuses as high-income countries with Indigenous minority populations.^21^

### Search strategy

A systematic search was conducted of the following databases: MEDLINE, EMBASE, CINAHL, Informit, Google Scholar, Circumpolar Health, and Australian Indigenous Health InfoNet. Unpublished studies were identified by searching the Agency for Healthcare Research and Quality (AHRQ), Google, iPortal, Native Health Database, New Zealand Government Ministry of Health, Australian Government Department of Health, Government of Canada Open Data Portal, Canada Health Infoway, Canadian Agency for Drugs and Technologies in Health (CADTH), and ClinicalTrials.gov.

For database search strategies, a combination of Boolean operators, truncations, and Medical Subject Headings (MeSH) were used, supported by an academic librarian (SC) and approved by the Executive Director of Health (TH) at Carrier Sekani Family Services, a First Nations-led health care society. For the Google and Google Scholar searches, one team member reviewed the first 50 relevancy-ranked results. The final search strategy for MEDLINE is presented in Table 1.

**Table 1.** Search strategy for MEDLINE.

Category	Search Terms
Population	1. exp Indians, North American/
	2. exp Alaska Natives/
	3. exp Inuits/
	4. exp Indigenous Peoples/
	5. exp Health Services, Indigenous/
	6. exp Oceanic Ancestry Group/
	7. ((Indigenous adj3 australia*) or (aborigin* adj3 australia*) or Torres Strait Islander* or Maori or Tribe or Tribes or Tribal).mp.
	8. (Indigenous or First Nation* or Inuit* or Metis or Aborigin* or (Native* adj3 America*) or American Indian* or (America* adj3 Native*) or Amerind* or (Alaska* adj3 Native*)).mp.
	9. 1 or 2 or 3 or 4 or 5 or 6 or 7 or 8
Concept	10. exp Telemedicine/
	11. exp Telemetry/
	12. exp Telenursing/
	13. exp Telerehabilitation/
	14. exp Mobile Applications/
	15. exp Smartphone/
	16. exp Cell Phone/
	17. exp Biometry/
	18. exp Biometric Identification/
	19. exp Wearable Electronic Devices/
	20. exp Biosensing Techniques/
	21. exp Self-help Devices/
	22. exp Monitoring, Physiologic/
	23. (telehealth* or tele-health* or telemedicine or tele-medicine or telecare or tele-care or telenurs* or tele-nurs* or mobile health* or ehealth or e-health or mhealth or m-health or telemonitor* or tele-monitor* or telerehabilitat* or tele-rehabilitat* or remote medicine or remote health* or distance medicine or digital health* or remote biometr* or (remote adj2 monitor*) or (virtual adj2 care) or teledermatology or tele-dermatology or telepsychiatry or tele-psychiatry or teleophthalmology or tele-ophthalmology or teleneurology or tele-neurology or telecardiology or tele-cardiology or telerheumatology or tele-rheumatology or teleoncology or tele-oncology or teleorthopedics or tele-orthopedics or telepneumology or tele-pneumology or telecommunication or tele-communication).mp.
	24. (wearable* or smart device* or health sensor* or health monitor* or biosensor* or biometric* or mobile technolog* or mobile monitor* or smartphone* or smart phone* or cellphone* or cell phone* or mobile phone* or app or apps or fitbit* or fitness tracker*).mp.
	25. 10 or 11 or 12 or 13 or 14 or 15 or 16 or 17 or 18 or 19 or 20 or 21 or 22 or 23 or 24
	26. 9 and 25
Context	Canada, the United States, Australia, New Zealand (applied at the full text review stage)

### Eligibility criteria

To be included, documents had to focus on the use of telehealth in Indigenous populations in Australia, Canada, New Zealand, and the US and be available between Jan 1, 2000 and May 2, 2022. “Telehealth” was considered to be health care whereby healthcare practitioners (HCP) interact with their patients through technology, including video, audio, messaging, the internet, “apps,” or “wearables.” Indigenous people were required to represent at least 50% of participants included in the studies. Selection was restricted to English language documents, due to linguistic abilities of the study team. Only original research papers were included in the final analysis, but there was no restriction on study design.

### Study selection

**Figure 1.**
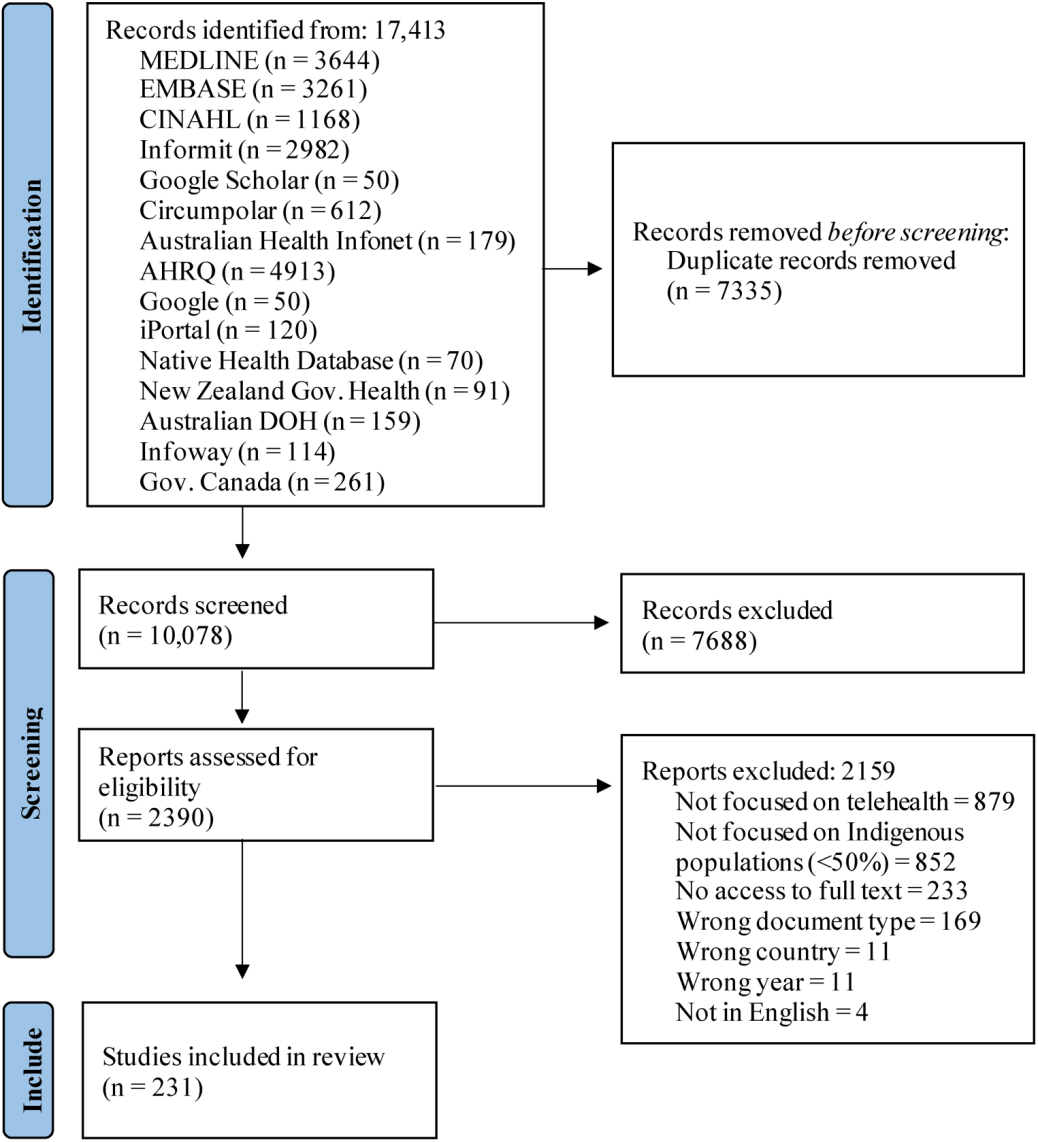
PRISMA flowchart of identification and selection of studies process.

Following the search, all identified citations were uploaded into Covidence (www.covidence.org, 2019, Veritas Health Innovation Ltd, Melbourne, Australia) or Microsoft Excel, and duplicates were removed. Two team members independently screened titles and abstracts, and a third team member solved any discrepancies. The same process was undertaken for the full text review. Reasons for exclusion are provided in Figure 1.

### Data extraction and analysis

Data extracted from included full-text documents were title, year of publication, author names and affiliation, study location, study design, keywords, study population, participants’ demographics, objectives of the study, inclusion/exclusion criteria, outcome measurements, involvement of Indigenous people, technology used, intervention (brief description, length, and who provided it), healthcare conditions being addressed, key concepts on effective use, cultural safety, and therapeutic relationships.

Descriptive statistics, including frequency counts and percentages, was calculated in Microsoft Excel (2019) to provide an overview of the breadth of the published and unpublished literature. A narrative synthesis was used to provide textual representation of results, supplemented by tables and graphics whenever appropriate. Qualitative data analysis was conducted to answer the sub-questions related to effective use, cultural safety, and therapeutic relationships. This process involved identifying emergent patterns, labeling codes to data, and combining these codes into overarching themes that meaningfully represent the data and respond to the research question.^27^

## Results

### Sources of evidence

A total of 17413 documents were identified after searching the selected databases and 7335 duplicates were excluded. After screening for 10,078 titles and abstracts, 7688 papers were excluded and 2390 were retrieved for full-text evaluation. A total of 231 records were included in this scoping review,^6,8,18–21,28–252^ The study selection process is outlined in Figure 1.

### Characteristics of studies

**Figure 2.**
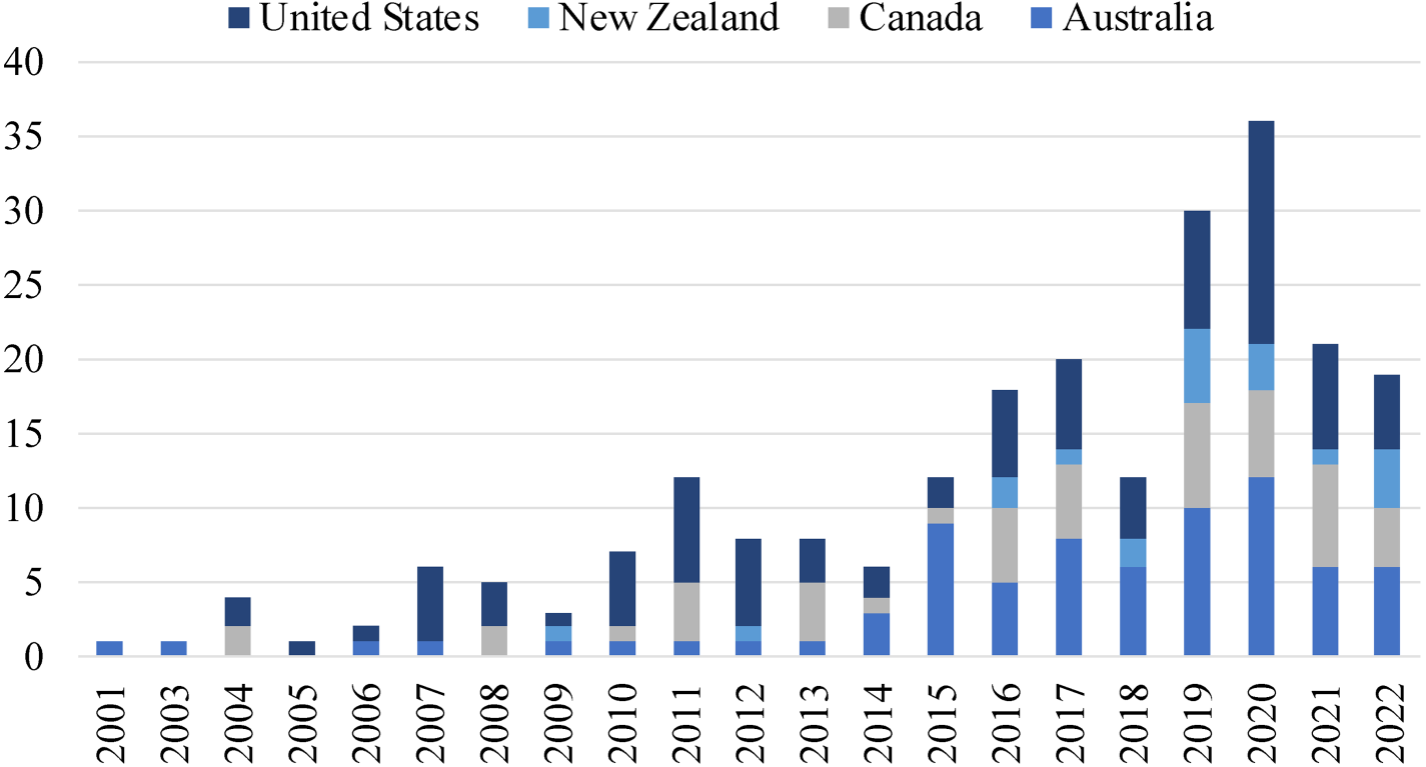
Timeline of publications per year (2000 to May 2022).

A complete table with the overarching characteristics of the final data set is available as a Supplementary file (Table S1). The majority (82%) of included papers were published in the last decade, and approximately two-thirds (60%) were published within the last 5 years (Figure 2). Thirty-eight percent of studies were from the United States, 32% from Australia, 21% from Canada, and 9% from New Zealand. Thirty-eight percent of papers used quantitative research methods, 27% applied qualitative research methods, 10% were reviews, 10% utilized a mixed-methods approach, 9% were program reports, and 6% were case reports. Regarding geographical setting, more than half of the studies were conducted in rural locations (51%). Six percent of studies focused on people from urban locations, 15% included both rural and urban populations, and 28% did not provide this information.

Across the studies that involved participants (e.g., were not reviews or program reports) and described their characteristics in terms of age range, 74% focused on adults, 41% on youth, 34% on seniors, and 17% on children. Across the studies that described participants’ biological sex, most included both males and females (85%), 9% focused only on females, and 6% focused only on males.

### Indigenous communities’ representation and involvement in the studies

**Figure 3.**
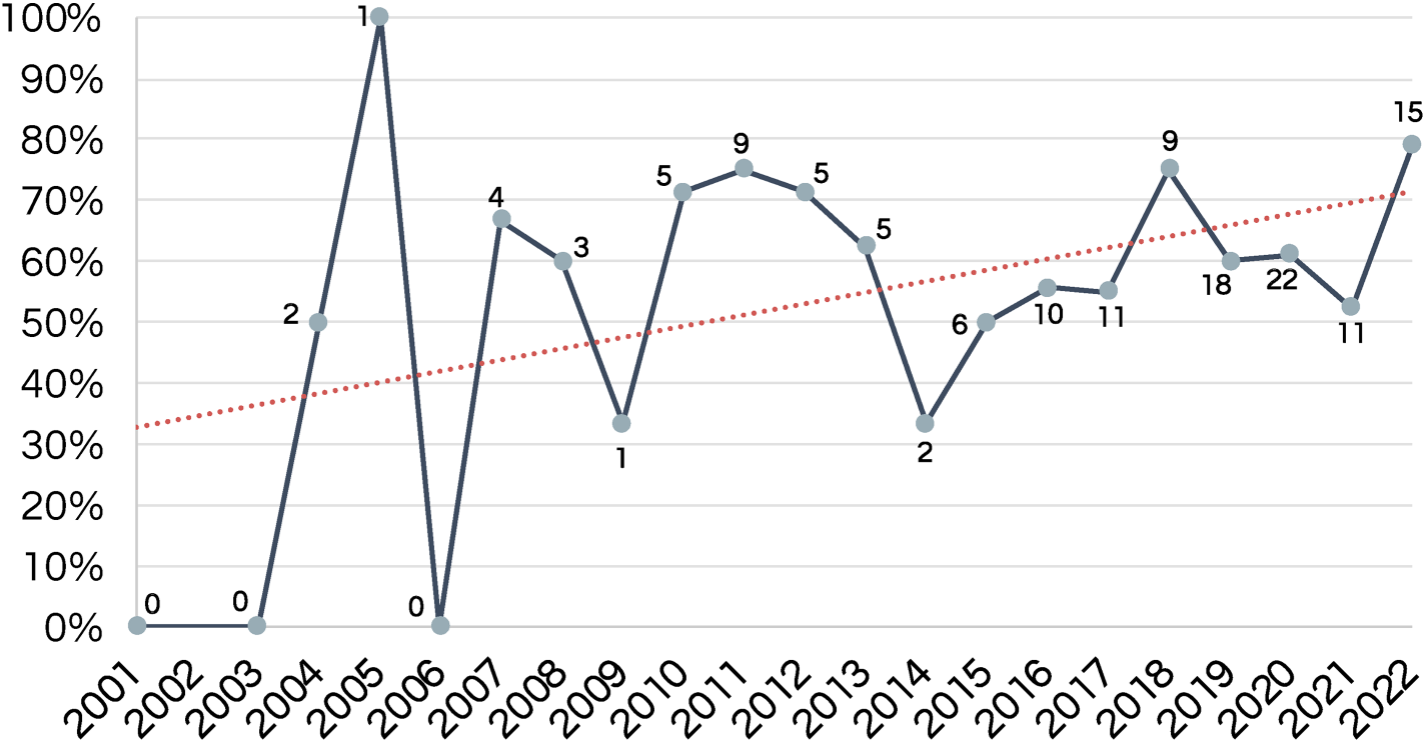
Proportion of studies that reported Indigenous involvement per year.

How Indigenous communities were identified in the studies differed across countries. Most authors used broad group names (e.g., Indigenous, Aboriginal), with only 11 Indigenous communities specifically named (Table 2). Although only 60% of the studies explicitly reported having Indigenous involvement in the development and implementation of telehealth services, the proportion of papers that reported Indigenous involvement increased over time—being more frequent in more recent publications (Figure 3).

**Table 2.** Indigenous groups and how they were identified in the studies.

Country	Studies (n)
Australia	
Indigenous or Aboriginal (unspecified)	29
Torres Strait Islander	45
Canada	
Indigenous or Aboriginal (unspecified)	14
First Nations	28
Inuit	14
Métis	8
New Zealand	
Indigenous or Aboriginal (unspecified)	1
Māori	19
Pacific Islanders	8
United States	
Indigenous, Native American, American Indian (unspecified)	75
Alaska Native/Alaska Inuit	45
Anishinaabe	2
Native Hawaiian	4
Pacific Islander	5
Navajo	2

### Type of technology

The largest percentage of studies (44%) utilized mobile health (mHealth) services, which included text messages and mobile applications (apps). Videoconference was the second most utilized telehealth technology (25%). Web-based interventions, which included websites and use of social media channels, were applied in 20% of the studies. Store-and-forward (12%), remote patient monitoring (8%), others (including CD, TV, radio, and video) (5%), electronic health record (3%), and accelerometer (2%) were less commonly utilized. Table S1, column 6, shows the detailed information for this analysis.

### Health focus of the telehealth interventions

Telehealth interventions for Indigenous people focused on a variety of health problems, but mental health (26%) was the most common, followed by diabetes and diabetic retinopathy (13%), ear, nose, and throat diseases (8%), cigarette smoking (6%), alcoholism (5%), and cancer (5%). Some studies developed interventions to promote a healthy lifestyle, without focusing on any particular disease (5%), and a few studies targeted chronic conditions without specifying any disease (4%). All other conditions listed represented 3% or less of the overall included studies. Table S1, column 7, shows the detailed information for this analysis.

### Key concepts for effective use

A number of barriers to the effective use of telehealth by Indigenous communities were identified by 54% of included papers. Table 3 highlights each barrier and facilitator, with the proportion of papers that discussed it and the respective citation number. The three most frequently-reported barriers were: 1) concerns about privacy and confidentiality, 2) limited broadband internet availability, and 3) low health/digital literacy (lack of confidence and skills to use the proposed technology for health purposes). Both patients and HCP raised concerns about the difficulty to enable and enhance trusting relationships due to the lack of physical touch and face-to-face interaction in telehealth settings (17%). Communication challenges (such as difficulty to pick up nonverbal cues, low number of service providers speaking Indigenous languages, and relatively few available interpreters), technical difficulties with the technology (such as audio delay and image freezing), and reduced access to the equipment (often associated with the costs of buying a smartphone, tablet, and computer) impacted Indigenous people experiences with telehealth care and were each discussed in approximately 16% of the studies. Some studies reported the perception from HCP about telehealth services being labor-intensive and requiring extra work time (10%). Staffing difficulties, mainly related to recruitment and retention of HCP and technicians to resolve problems with technology, were addressed by 9% of studies. Lack of funding and reimbursement issues were also commonly reported (9%). Comments about insufficient training and criticism on the inability to perform physical examination (which directly impairs safety in acute cases that require immediate attention) were each seen as barriers by 8% and 7% of the studies, respectively. Less common were negative comments about content and aesthetics (5%), which are likely related to lack of cultural adaptation (4%).

**Table 3.** Barriers and facilitators for telehealth effective use by Indigenous populations.

Barriers for Effective Use	Proportion of studies (%)	References
Concerns about privacy and confidentiality	18	^8,18,33,46,65,76,78,80,93,100,107,108,130,143–146,173,183,208,225,227,231,240,245,247,248^
Limited broadband internet availability	18	^8,21,61,76,84,85,107,110,111,117,132,133,146,151,160,173,187,189,208,220,223,225,229,231,235,238,240^
Low health/digital literacy	18	^19,35–37,41,61,77,87,108,120,124,125,132,167,173,177,182,187,189,205,215,235,242–245^
Difficulty to enable and enhance trusting relationships	17	^8,18,33,35,61,65,87,93,95,114,137,139,154,175–177,183,187,191,194,196,208,216,231,234^
Communication challenges	16	^18,39,93,95,109,110,131,135,141,149,152,154,161,170,173,177,185,189,194,223,231,239,243,248^
Technical difficulties with the technology	16	^33,34,39,73,76,88,91,93,95,109,112,120,137,154,160,167,169,173,176,194,198,231,240^
Reduced access to the equipment	16	^8,33,54,61,66,70,88,108,135,137,146,150,154,155,171,177,182,222,225,235,242,243^
Labor-intensive and require extra work time from health care providers	10	^8,58,65,89,110,114,124,170,176,177,179,185,187,189,215^
Staffing difficulties	9	^39,82,107,109,124,151,167,169,171,176,199,202,216^
Lack of funding and reimbursement issues	9	^39,89,100,117,135,143,169,170,176,185,189,220,243,244^
Insufficient training	8	^41,61,70,91,114,131,167,173,175,187,220,231^
Inability to perform physical examination (risk for safety)	7	^71,85,91,93,109,126,136,144,154,174,212^
Negative comments about content and aesthetics	5	^21,99,152,166,222,227,248^
Lack of cultural adaptation	4	^71,93,109,126,136,212^
Facilitator for Effective Use	Proportion of studies (%)	References
Cultural relevance and appropriateness	29	^8,19,21,32,40,46,51,52,57,58,61,63,67,81,85,94,107,110,113,116,118,120,123,124,128,135,140,149,150^, ^152,161,164,169,175,182,184,195,225,231,232,240,242,248^
Community engagement	27	^30,39,52,58,65,78,79,81,95,97,99,104,107,111–113,116,117,127,130,138–140,145,146,148,150,151,154,159,164,169,170,173,183,189,193,202,214,215,226,229,233,234,245,248^
Convenience	24	^19,32,33,45,46,70,76,78,85,88,93,95,97–102,105,106,108,109,114,126,136,143,164,176,184,189,191,194,208,213,214,219,220,227,231,243,249^
Physical and financial accessibility	22	^19,33,38,45–48,70,71,76,78,8,82,93,95,98,101,103,105,114,115,117,118,124,135,150,156,157,162–164,187,189,195,203,216,220,235,249^
Ability to enhance social support networks	16	^8,33,46,55,64,95,107,108,112,125,127,136,148,158,164,170,173,177,181,185,194,198,219,229,231,232,234,235^
Visually appealing, innovative, interactive, and fun technologies	15	^21,36,56,61,78,80,94,99,112,120,146,152,161,173–175,187,222,223,225,232,233,235,248^
Prior experience with technology and digital literacy	14	^54,61,87,89,90,94,104,107,120,135,137,169,173,177,178,205,225,229,231,233,235,237,240,248^
The timely nature of telehealth	13	^8,19,34,37,48,71,76,80,87,89,108,109,114,125,130,140,163,185,187,188,214,223^
Indigenous health care providers and cultural liaisons involved in the delivery of telehealth	13	^19,32,39,65,66,88,104,108,109,135,139,152,169,173,214,215,217,226,229,231,242^
Appropriate training	12	^6,19,35,37,39,41,61,72,94,107,110,113,117,127,133,177,179,189,193,199^
Good audiovisual quality	9	^28,38,45,49,64,82,84,126,143,162,191,197,210,212,219,240^
Ability to monitor your own health	9	^89,99,100,114,139,140,148,169,182,185,187,234,236,237,243^
Anonymity that some services warranted	6	^33,87,92,108,120,132,174,189,200,205,227^
Improved coordination of services	4	^145,151,167,206,214,231^

Studies also reported facilitators for effective telehealth use. Most importantly was the cultural relevance and appropriateness of telehealth programs, discussed in 29% of papers. This was seen to be achieved by tailoring the intervention to the targeted Indigenous group, co-designing and culturally adapting instruments, and integrating cultural content and practices (e.g., traditional healing, language, Indigenous graphics symbols, and design) to telehealth services. Studies stated that culturally relevant and appropriate telehealth services could not occur without community engagement, which was the second most frequently reported facilitator (27%). Consultation with community advisory boards and relationship building with community partners was considered fundamental.

The convenience (e.g., less travel time and safe environment) (24%), physical and financial accessibility (22%), and telehealth ability to enhance social support networks (16%) by, for example, enabling the attendance of family members at a person's consultations and avoiding disruption from family and broader community, positively impacted its acceptability and effectiveness. Visually appealing, innovative, interactive, and fun technologies facilitated Indigenous people's engagement (15%). Fourteen percent of studies reported that having prior experience with technology and digital literacy facilitated its effective use. The timely nature of telehealth was considered an advantage that favors its utilization (13%). Having Indigenous HCP and cultural liaisons involved in the delivery of telehealth facilitates uptake (13%), and providing appropriate training helps ensure local capacity building and facilitates successful implementation of telehealth (12%). In addition, things that were not so commonly mentioned include the good audiovisual quality of the videoconferences and good image quality of exams (9%), the ability for a patient to monitor their own health (9%), the anonymity that some services warranted (6%), and the improved coordination of services (4%).

### Key concepts for cultural safety

Cultural safety was discussed to some extent in 174 studies (75%). Table 4 highlights each key concept, with the proportion of papers that discussed it and the respective citation number. The most commonly reported (48%) way to achieve cultural safety was involving Indigenous communities in the development and implementation of telehealth services, working in partnership and close collaboration with Indigenous people, iteratively consulting with Indigenous advisory boards, and building lasting relationships with Indigenous partners.

**Table 4.** Key concepts for cultural safety in telehealth services for Indigenous populations.

Key Concepts for Cultural Safety	Proportion of studies (%)	References
Indigenous communities’ involvement	48	^20,21,30,33,35,37–40,43,46,50,53,54,56,60,69,70,80,97,99,100,103,107,108,111,112,114–116,126,127,130,132,134,136,138–140,145,146,148–150,152,155,159,164,166,167,170,173–175,181,184,189,190,193,198,202,206,208,213–215,223,226–231,232,233,238,239,241,242,245,251^
Tailor telehealth to the Indigenous groups it is targeted toward	32	^6,20,40,43,50,51,54,56,60,61,66,72,79,99,103,111,114,120,125,132–134,136,138,139,146,149,159,164,167,172,174,184–187,189–192,195,197,198,202,203,205,206,214,222,225,228,237,238,244,247,248^
Respect for Indigenous cultural values	30	^8,18,32,34,35,37,47,54,63,79,88,89,97,100,103,109,110,112,118–120,123,128–130,133,139,148,149,151,155,158,161,166,170,172,173,179,182,190,191,200,215^, ^225,230,233,234,238,240,241,245^
Use of Indigenous methodologies, strength-based approach, and appropriate ethics procedures in research	21	^8,29,32,35,41,53,54,63,69,76,80,88,107,110,116,131,139,149,155,166,179,192,197,223,226,228,230–235,239,243,246,250^
Cultural competence and cultural safety training	12	^18,37,46,65,79,93,95,107,131,149,177,188,194,205,219,233,245^
Acknowledge the influences of historical, economic, and social factors on Indigenous people healthcare experience	13	^34,40,46,48,65,79,85,88,91,93,95,103,106,116,128,145,148,152,175,222,229,241^
Honour Indigenous sovereignty and self-governance	6	^48,51,53,65,97,164,175,198,227,243^
Have Indigenous health care workers facilitate the delivery of intervention	9	^88,90,107,120,126,128,135,140,149,155,166,174,181,184,217^
Work in partnership with cultural liaisons	6	^51,65,88,149,150,152,159,170,219,226,239^

Telehealth services were noted as needing to be tailored to the Indigenous groups to whom they are targeted (32%). One example of this tailoring step was provided in the paper by Vigil-Hayes et al.,^238,239^ who created the ARORA mobile app which was designed to support social and emotional learning interventions for Navajo youth. The research team included a researcher who is Navajo and was able to assist in the initial conceptual design of intervention, creating learning activities that used culturally-significant images and concepts that are particularly relevant to Navajo people.^238,239^ In another study^184^ a website entitled the Keya Tracker was designed to support the control of Type 2 diabetes among Indigenous people from a Northern Plains Indian reservation. Community tribal elders were involved in the design of the title, colors, and graphics, and content.^184^ A third example was the study that reported on “OL@-OR@,” a mHealth program designed to support healthy lifestyles in Māori and Pasifika peoples.^79^ It contained culturally relevant content, such as activity groups specifically for Māori and Pasifika, promotion of wellbeing through atua (gods) concepts, tikanga (Māori customs), blessings, culturally relevant recipes, whakatauki (Māori proverbs), and cultural icons and imagery used as virtual rewards when participants achieved their goals.^85^

Respect for Indigenous cultural values, demonstrated through the incorporation of cross-cultural conceptualizations of health and wellness, traditional healing practices, language, and ceremonies (30%) helped improve cultural safety. In a research process, the use of Indigenous methodologies (e.g., storytelling and sharing circles), strengths-based approaches that shift the perceived deficits away from the individual and place health problems in the appropriate context, and following Indigenous protocols and ethics procedures were strongly recommended steps toward cultural safety in research (together represented in 21% of studies).

Acknowledging the influences of historical, economic, and social factors on Indigenous people's healthcare experiences (13%), completing cultural competence and cultural safety training (12%), and honoring Indigenous sovereignty and self-governance (6%) were also recommended to promote cultural safety. Finally, having Indigenous HCP (9%) and cultural liaisons (6%) facilitating telehealth interactions was believed to increase cultural safety.

### Key concepts for building therapeutic relationships

Sixty-four studies talked about the therapeutic relationships in telehealth. Among these, 38% viewed telehealth as a way to improve the continuity and consistency of the therapeutic relationships, mainly by improving the frequency of contact and providing a sense of connection.^8,18,33,39,41,45,46,61,70,76,78,85–89,92,95,97,101,103,107,109,114,125,131,137,139,143,144,154,158,160,163,169,173–177,183,185,187,189,191,193,194,196,198,200,205,208,209,214,216,217,227,231,234,242,243,248^ In one study, the Stay Strong App, designed to addresses mental health and wellbeing concerns of First Nations Australians, was described by service providers as a good “leveler,” equalizing the power imbalance often present in their relationships with clients.^61^ One participant said it would help changing the perception of his role from a “monitor and accountability person” to a partner in making change.^61^ A few studies reported improvements in cross-cultural communication in telehealth,^89,176,227^ and others talked about how interpersonal distance in telehealth facilitates the disclosure of sensitive matters.^33,92,198,200^ Both aspects were considered critical to establishing trust and rapport between patient and HCP.

Conversely, many studies (39%) reported Indigenous preferences for face-to-face interactions and raised concerns about the depersonalization of the therapeutic relationship in telehealth.^8,33,61,86,87,95,139,154,175–177,187,191,194,196,208,216,231,234^ Studies reported patients having difficulty building trust over video, taking longer to establish rapport, and feeling overall uncomfortable seeing an HCP over video.^86,87,139,154,194,216,231^ Reasons for this varied, but included technical issues making the conversation less personable, concerns that the session becomes about the technology rather than the engagement, and communication issues due to the lack of nonverbal cues.^95,176,187,191,196^

Seventeen studies (27%) suggested that a potential way to address this issue, at least partially, would be having the first visit in-person.^18,33,76,86,88,101,103,109,114,154,158,173,183,193,198,217,248^ Having telehealth outreach workers was seen as a strategy to create a connection between Indigenous clients and urban HCP (9%), to reduce cultural barriers and provide community context.^39,46,70,214,231,242^ Finally, cultural safety training and local capacity building focused on telehealth training was seen to support the development of positive therapeutic relationships in telehealth (8%).^88,131,163,174,243^

## Discussion

The primary aim of this review was to map and characterize the available evidence on telehealth used by Indigenous people in Australia, Canada, New Zealand, and US. Contrasting with other reviews,^6,19,20^ we did not find a paucity of studies on telehealth for Indigenous people, and our findings showed a steady increase in the number of publications in the last 4 years indicating a growing field of research.

The most important finding of this review is that although community engagement was the most frequently reported way to achieve cultural safety, and the second most reported facilitator for telehealth effective use by Indigenous people, only 60% of the studies explicitly mentioned having Indigenous involvement in the development and/or implementation of telehealth services and/or the research process. This gap that was previously identified by Fraser et al.^18^ who commented on the lack of research from a cultural safety perspective. According to the United Nations Declaration on the Rights of Indigenous Peoples (UNDRIP), article 23, Indigenous peoples have the right to be actively involved in developing and determining health program affecting them.^253^ In New Zealand, for example, the assertion of tino rangatiratanga in health policies provided space for Māori cultural practices in health services. Tino rangatiratanga, as established by the 2019 Hauora report, is a principle that recognizes that Māori is both a legitimate and an essential part of decision-making in the health and disability sector, and provides for Māori self-determination in the design, delivery, and monitoring of health and disability services.^254^

Furthermore, practices that support cultural safe care for Indigenous people are effective communication, understanding and taking into consideration how historical, economic, and social factors shape Indigenous health, and willingness to integrate Indigenous beliefs, practices, identity, and cultural background into the treatment plan.^255^ Our findings suggest that all these things can be facilitated through telehealth, but they can also end up being barriers if telehealth is not thoughtfully planned. Thus, continued self-reflection from health care providers (i.e., how do you see your cultural backgrounds influencing your communication and treatment), and training on cultural competence/safety, trauma-informed care, traditional practices, and how to use the technologies to their full extent accounting for all these things is required.^256^

Telehealth is still often linked to the use of videoconferencing. However, new technologies have emerged, and research has started to investigate its potential role in addressing health disparities experienced by Indigenous groups.^20,21,257^ Our review showed that mHealth was the most frequently reported modality in the studies. In particular, mobile applications were explored by 68 studies. Among these, all studies that assessed acceptability demonstrated overall positive perceptions. Importantly, El Sayed et al. noted that “for new information to be accepted by Aboriginal societies, the process can be just as important as the content.”^70^ Therefore, co-design was the recommended approach to deliver sustainable, efficient, and culturally appropriate telehealth programs that would be acceptable to their target audience. Specific aspects to increase acceptability and uptake include visual appeal (e.g., use of Indigenous art, colors, and language), ease of use, innovative format, apps that were interactive and fun, and cultural relevance of its content. Fifteen studies used telephone in conjunction with another technology.^6,29,39,47,51,71,105,166,171,179,187,189,195,208,211^ Of note, we excluded five studies that exclusively used telephone calls as their telehealth technology. We do not think that adding these papers would have contributed to significant changes in our findings, but we acknowledge telephone may be a viable and inexpensive technology for some communities.

With respect to the health focus of the interventions, 58 studies described telehealth interventions that targeted primarily mental health. As a group, Indigenous populations across the globe experience a disproportionate burden of mental illness, which have been consistently associated with Indigenous experiences of colonization or historical trauma.^258^ A variety of technologies and telehealth modalities have been used to tackle mental health problems, but 47% used mHealth and 40% used videoconference. Generally, participants from the studies with a focus on tele mental health reported high acceptance levels. Many Indigenous people have reported feelings of “shame” in accessing mental health services, so mHealth can help overcome this barrier ensuring anonymity and privacy.^87,174,227^ On the other hand, Russel et al.^191^ argue that a positive aspect of videoconferencing is the physical aspects of it, including being able to see and hear the person clearly on the video.

Research supports the vital role of strong relationships mediating Indigenous peoples’ health and wellness, and a strong therapeutic relationship is a critical contributor to positive health outcomes for Indigenous peoples.^259–261^ A major finding of this review is that many studies raised concerns about the difficulty to enable and enhance trusting relationships in telehealth because of reduced human connection due to the lack of face-to-face interaction. On the other hand, several studies also reported positive perceptions of telehealth increasing consistency and continuity of these relationships. Factors behind these contrasting experiences vary, but a suggested way forward was having the first visit being in-person, to facilitate the bond forming, and then follow-up visits via telehealth. Hybrid programs have the potential to be more effective than exclusively virtual ones.^262^ Other factors that facilitate building trusting relationships in telehealth are cultural safety training by the HCPs and local capacity building.^88,131,163,174,243^ Telehealth can accommodate the presence of Indigenous health workers, and this is strongly recommended since they are able to provide a better understanding of the community members’ personal circumstances to the specialists, facilitate communication, and empower Indigenous patients to make decisions about their care, in addition to building their own capacity.^13,53^ Understanding that telehealth is not the best model of healthcare delivery for all patients and considering each patient's personal preferences is also essential to patient-centered care and prioritizing strong therapeutic relationships.^263^

There are noteworthy limitations in this review. We limited our eligibility criteria to papers published between 2000 and 2022, written in English, and were original research papers. This was a choice for the feasibility of completing the review, but we acknowledge that relevant studies may have been excluded due to this criterion. In addition, we limited our review to four English-speaking countries due to their similar colonial history, however, we recognize that many other Indigenous nations exist, and our findings cannot generalize to other countries and Indigenous populations.

## Conclusion

There is a large and growing body of literature on the use of telehealth by Indigenous populations in Australia, Canada, New Zealand, and the US. Telehealth technologies are not an all-encompassing solution to the various factors impacting Indigenous people's health. We identified many barriers to its effective use, such as privacy and confidentiality issues, limited broadband internet availability, and low health and digital literacy. Nevertheless, when developed and implemented in close collaboration with the communities, telehealth services are a promising way to facilitate the provision of culturally appropriate healthcare for Indigenous populations.

## Supplemental Material

sj-docx-1-jtt-10.1177_1357633X231158835 - Supplemental material for Scoping review of telehealth use by Indigenous populations from Australia, Canada, New Zealand, and the United States Supplemental material, sj-docx-1-jtt-10.1177_1357633X231158835 for Scoping review of telehealth use by Indigenous populations from Australia, Canada, New Zealand, and the United States by Débora Petry Moecke, Travis Holyk, Madelaine Beckett, Sunaina Chopra, Polina Petlitsyna, Mirha Girt, Ashley Kirkham, Ivan Kamurasi, Justin Turner, Donovan Sneddon, Madeline Friesen, Ian McDonald, Nathan Denson-Camp, Stephanie Crosbie, and Pat G. Camp in Journal of Telemedicine and Telecare
