# Supplementary material for: Scoping review of telehealth use by Indigenous populations from Australia, Canada, New Zealand, and the United States
Source: J Telemed Telecare. 2023 Mar 13;30(9):1398–416. doi: 10.1177/1357633X231158835 (PMC11411853; doi:10.1177/1357633X231158835)
Supplement: sj-docx-1-jtt-10.1177_1357633X231158835 - Supplemental material for Scoping review of telehealth use by Indigenous populations from Australia, Canada, New Zealand, and the United States [file sj-docx-1-jtt-10.1177_1357633X231158835.docx]

**Supplementary files.**

**Table S-1.** Overarching characteristics of studies.

| **Study (main author, year)** | **Country** | **Study Design** | **Participants demographics**  **(age range, sex)** | **Indigenous involvement** | **Type of technology** | **Health focus** |
| --- | --- | --- | --- | --- | --- | --- |
| Abbas 2008 (28) | United States | Cohort study | All ages, both sexes | No | Store-and-forward | Rheumatic heart disease and acute rheumatic fever. |
| Albright 2012 (29) | United States | Randomised controlled trial | Adulthood (25-64), women | No | - Telephone - Accelerometer | Physical activity |
| Anastario 2020 (30) | United States | Qualitative research | Youth (15-24), both sexes | Yes | Laptop computers using computer assisted self-interview (social media use was one of the topics of investigation) | Sexual risk behaviours |
| Anderson 2017 (31) | New Zealand | Case report | Childhood (0-14 years) and Youth (15-24), both sexes | No | ActiGraph wGT3X-BT (ActiGraph, Pensacola, Florida, USA) accelerometer | Physical activity and sedentary behavior of overweight/obese children. |
| Arora 2013 (32) | Canada | Mixed methods | NR | Yes | Store-and-forward | Ocular pathology, especially diabetic retinopathy. |
| Babbage 2020 (33) | New Zealand | Qualitative research | Youth (15-24), both sexes | No | In this study, technology has been broadly defined and includes telehealth, e-Health, and m-Health | Overall health |
| Barsky 2019 (34) | Canada | Mixed Methods | Youth (15-24), Adulthood (25-64), and Seniority (65 years +), both sexes | Yes | Short Message Service (SMS) text messaging–based system | Uncontrolled hypertension |
| Bennett-Levy 2017 (35) | Australia | Qualitative research | Adulthood (25-64), both sexes | Yes | e-Mental Health (e-MH):  - YouTube video clips  - Mobile applications (apps) | e-MH uptake amongst Aboriginal and Torres Strait Islander health professionals. |
| Bethune 2009 (36) | New Zealand | Mixed Methods | Adulthood (25-64), women | Yes | - Television, radio, and print media advertisements  - Telephone | Cervical cancer |
| Boushey 2016 (37) | United States | Cohort study | NR | Yes | Mobile app | Amnesic shellfish poisoning due to domoic acid exposure. |
| Brazionis 2018 (38) | Australia | Cross sectional study | Youth (15-24), Adulthood (25-64), and Seniority (65 years +), both sexes | Yes | - Retinal image scanning technologies - Electronic health record | Diabetes |
| **Study (main author, year)** | **Country** | **Study Design** | **Participants demographics**  **(age range, sex)** | **Indigenous involvement** | **Type of technology** | **Health focus** |
| Brooks 2012 (39) | United States | Qualitative research | Adulthood (25-64), both sexes | Yes | - Telephone used for interviews in the study itself - Discussion in interviews of "traditional videoconferencing" vs "home-based telehealth services" | Post-Traumatic Stress Disorder (PTSD) |
| Brooks 2013 (40) | United States | Case report | Adulthood (25-64), both sexes | Yes | Remote patient monitoring | PTSD symptomology alone or in combination with other psychiatric disorders. |
| Brown 2020 (41) | Australia | Qualitative research | Youth (15-24), Adulthood (25-64), and Seniority (65 years +), both sexes | Yes | Mobile app for training Indigenous community gatekeepers on suicide intervention skills | Suicidal ideation |
| Brusse 2014 (42) | Australia | Scoping review | NR | No | - Social media (on any platform) or mobile phone software or features (excluding voice features) - Any app that appeared to be aimed at an Indigenous audience or which reported intentions in that regard was included | Smoking cessation, sexual health, and otitis media. |
| Burhansstipanov 2018 (43) | United States | Program report | Adulthood (25-64) and Seniority (65 years +) | Yes | - Cell phones as part of mHealth protocol (phone call and text only)  - iPads used for the informed consent process and data collection | Cigarette smoking |
| Bursell 2018 (44) | United States | Cross sectional study | Adulthood (25-64), both sexes | No | Retinal image scanning technologies | Diabetes |
| Caffery 2017 (45) | Australia | Systematic review | NR | No | - Store-and-forward - Videoconference - Remote telehealth monitoring | Multiple conditions including mental health, oncology, palliative care, and anaesthetics. |
| Caffery 2018 (46) | Australia | Qualitative research | NR | Yes | Videoconference | NR |
| Cardona 2022 (47) | Australia | Scoping review | NR | No | - Telephone  - Videoconference  - Store-and-forward | Multiple conditions including mental health, diabetes, chronic kidney disease, heart failure, and prostate cancer. |
| **Study (main author, year)** | **Country** | **Study Design** | **Participants demographics**  **(age range, sex)** | **Indigenous involvement** | **Type of technology** | **Health focus** |
| Carroll 2011 (48) | United States | Program report | NR | Yes | - Electronic health record  - Store-and-forward | - Ear, nose, and throat diseases  - Diabetic retinopathy |
| Cartwright 2015 (252) | Australia | Program report | Adulthood (25-64) and Seniority (65 years +), both sexes | No | Remote patient monitoring | Chronic health conditions (the most common were diabetes, hypertension or hypotension). |
| Chin 2014 (49) | United States | Cross sectional study | All ages, both sexes | No | Retinal image scanning technologies | Diabetic retinopathy |
| Choukou 2021 (19) | Canada | Scoping review | Seniority (65 years +) | No | Telehealth | Chronic disease and other age-related health disorders |
| Christie 2019 (50) | New Zealand | Qualitative research | Youth (15-24) | Yes | Mobile app | Mental health |
| Couch 2021 (51) | Australia | Cohort study | NR | No | - Telephone  - Videoconference | Chronic disease management |
| Craig Rushing 2021 (52) | United States | Randomised controlled trial | Youth (15-24), both sexes | Yes | SMS | Mental health |
| Darroch 2016 (53) | Canada | Qualitative research | Youth (15-24) and Adulthood (25-64), women | Yes | Mobile app | Pregnant or postpartum women |
| Davies 2015 (54) | Australia | Qualitative research | Adulthood (25-64), both sexes | Yes | Mobile app | Chronic hepatitis B |
| Dawson 2020 (6) | United States | Systematic review | Youth (15-24), Adulthood (25-64), and Seniority (65 years +) | No | - Telephone based - Web-based - SMS - Video | Multiple healthcare conditions including hypertension and diabetes |
| Dawson 2021 (55) | Canada | Cohort study | Adulthood (25-64) and Seniority (65 years +), both sexes | Yes | Mobile Diabetes Telemedicine Clinics | Diabetes |
| Dellinger 2018 (56) | United States | Qualitative research | Adulthood (25-64), both sexes | Yes | Mobile app "Gigiigoo’inaan" [Our Fish] | Food poisoning for inadequate fish consumption (contaminants) |
| Dellinger 2022 (57) | United States | Randomised controlled trial | Youth (15-24), Adulthood (25-64), and Seniority (65 years +), both sexes | Yes | Mobile app “Gigiigoo’inaan” (Our Fish in Ojibwe) | Food poisoning for inadequate fish consumption (contaminants) |
| **Study (main author, year)** | **Country** | **Study Design** | **Participants demographics**  **(age range, sex)** | **Indigenous involvement** | **Type of technology** | **Health focus** |
| Devan 2021 (58) | New Zealand | Qualitative research | NR | Yes | Online pain management programme (iSelf-help) | Pain management |
| Dick 2007 (59) | United States | Program report | Adulthood (25-64) | Yes | - Website  - Interactive CD-ROM  - Videos | Multiple conditions including alcohol and other drugs use, hepatitis C, birth control methods, diabetes, and stress management |
| Dignan 2019 (60) | United States | Randomised controlled trial | Youth (15-24), Adulthood (25-64), and Seniority (65 years +), both sexes | Yes | mHealth education and support through SMS | Cigarette smoking |
| Dingwall 2015 (61) | Australia | Qualitative research | Adulthood (25-64), both sexes | No | Stay Strong Mobile app | Health worker continuing education (mental health) |
| Dingwall 2015 (62) | Australia | Qualitative research | Youth (15-24), Adulthood (25-64), and Seniority (65 years +), both sexes | Yes | Aboriginal and Islander Mental Health Initiative (AIMhi) Stay Strong app | Health worker continuing education (mental health) |
| Dingwall 2021 (63) | Australia | Randomised controlled trial | Adulthood (25-64), both sexes | Yes | AIMhi Stay Strong app and The Hep B Story | End-stage kidney disease and chronic kidney disease |
| Doorenbos 2010 (64) | United States | Cross sectional study | Adulthood (25-64), women | Yes | Videoconference | Cancer |
| Doorenbos 2011 (65) | United States | Program report | NR | Yes | - Videoconference  - Store-and-forward tele-dermatology consults | Cancer |
| Dotson 2017 (66) | United States | Cross sectional study | Adulthood (25-64), both sexes | Yes | Computers and mobile phones | Smoking |
| Downs 2008 (67) | Canada | Cross sectional study | Childhood (0-14 years), both sexes | Yes | Pedometers | Obesity |
| **Study (main author, year)** | **Country** | **Study Design** | **Participants demographics**  **(age range, sex)** | **Indigenous involvement** | **Type of technology** | **Health focus** |
| Duma 2020 (68) | United States | Cross sectional study | Adulthood (25-64), women | No | eHealth/Telemedicine | Breast cancer |
| El Sayed 2011 (69) | Australia | Case report | Adulthood (25-64), both sexes | Yes | CD-ROM interactive multimedia | Health worker continuing education |
| El Sayed 2013 (70) | Australia | Qualitative research | Adulthood (25-64), both sexes | Yes | CD-ROM interactive multimedia | Health worker continuing education |
| Ellis 2019 (71) | Canada | Cross sectional study | Childhood (0-14 years) and Youth (15-24), both sexes | No | - Videoconference  - Telephone | Mental health |
| Eriks-Brophy 2008 (72) | Canada | Qualitative research | Childhood (0-14 years) | No | Videoconference | Language disorders |
| Ferucci 2020 (73) | United States | Cross sectional study | Youth (15-24), Adulthood (25-64), and Seniority (65 years +), both sexes | No | Videoconference | Rheumatoid arthritis |
| Ferucci 2020 (74) | United States | Cross sectional study | Youth (15-24), Adulthood (25-64), and Seniority (65 years +), both sexes | No | Videoconference | Rheumatoid arthritis |
| Ferucci 2021 (75) | United States | Retrospective observational study | Adulthood (25-64), both sexes | No | Electronic health record, Videoconference | Chronic diseases |
| Ferucci 2022 (76) | United States | Mixed methods | Adulthood (25-64) and Seniority (65 years +), both sexes | Yes | Videoconference | Chronic diseases |
| Filiault 2009 (77) | Australia | Review | Age NR, men | No | Health call centers and Internet-based health information | Men’s health |
| Fiolet 2020 (78) | Australia | Qualitative research | Youth (15-24), Adulthood (25-64), and Seniority (65 years +), both sexes | Yes | Online/technological resources and tools | Family violence |
| **Study (main author, year)** | **Country** | **Study Design** | **Participants demographics**  **(age range, sex)** | **Indigenous involvement** | **Type of technology** | **Health focus** |
| Firestone 2020 (79) | New Zealand | Randomised controlled trial | All ages, both sexes | Yes | OL@-OR@ smartphone app | Reducing non-communicable diseases risk factors and develop healthy behavioural changes |
| Fleming 2019 (80) | New Zealand | Qualitative research | Youth (15-24), both sexes | Yes | Apps and websites | Mental health distress |
| Fletcher 2017 (81) | Australia | Qualitative research | Youth (15-24), men | Yes | Website and mobile-phone-based text-messaging and mood-tracker program | Mobile phone-based resources to support young Aboriginal fathers |
| Fonda 2020 (82) | United States | Economic evaluation | Adulthood (25-64) | Yes | Store-and-forward | Diabetic retinopathy |
| Fonda 2021 (83) | United States | Retrospective observational study | Youth (15-24), Adulthood (25-64), and Seniority (65 years +), both sexes | Yes | Retinal image scanning technologies | Diabetic retinopathy and diabetic macular edema |
| Fraser 2017 (18) | Australia | Systematic review | All ages, both sexes | No | The telehealth modalities described in the studies were mostly real-time videoconference, internet-based apps and portals, and asynchronous technologies | Chronic diseases including such conditions as cardiovascular, respiratory, and renal diseases |
| Friedman 2010 (84) | United States | Cohort study | Adulthood (25-64) and Seniority (65 years +), both sexes | No | Store-and-forward | Colorectal cancer |
| Gibson 2011 (85) | Canada | Qualitative research | Youth (15-24), Adulthood (25-64), and Seniority (65 years +), both sexes | Yes | Videoconference | Mental health |
| Gibson 2011 (86) | Canada | Qualitative research | Adulthood (25-64), both sexes | Yes | Telemental health | Mental health |
| Goodyear-Smith 2016 (87) | New Zealand | Mixed methods | Youth (15-24), both sexes | Yes | YouthCHAT - a youth program for electronic screening and intervention for lifestyle risk factors and mental health | Mental Health |
| Goss 2017 (88) | United States | Case report | Adulthood (25-64) and Seniority (65 years +), both sexes | Yes | Videoconference | Mental health |
| **Study (main author, year)** | **Country** | **Study Design** | **Participants demographics**  **(age range, sex)** | **Indigenous involvement** | **Type of technology** | **Health focus** |
| Gu 2016 (89) | New Zealand | Mixed methods | Youth (15-24), Adulthood (25-64), and Seniority (65 years +), both sexes | Yes | Electronic health record | Medication adherence to prevent cardiovascular disease risk |
| Gwynn 2020 (90) | Australia | Cross sectional study | Adulthood (25-64) and Seniority (65 years +), both sexes | Yes | A handheld smartphone electrocardiograph screening device | Atrial fibrillation |
| Habib 2022 (91) | Australia | Cohort study | Childhood (0-14 years), both sexes | No | Otoscopic images/ teleotology/ algorithms | Ear diseases (otitis media) |
| Hanson 2020 (92) | United States | Qualitative research | Youth (15-24), Adulthood (25-64), and Seniority (65 years +), both sexes | Yes | Web-based technology | Alcohol-exposed pregnancy |
| Haozous 2012 (93) | United States | Cross sectional study | NR | Yes | - Videoconference  - Electronic health record | Cancer-related pain |
| HealeyAkearok 2020 (94) | Canada | Mixed methods | NR | Yes | Mobile app | Orientation app for new and short-term health care providers in Nunavut |
| Hensel 2019 (8) | Canada | Review | NR | No | - Videoconference - Store-and-forward  - Electronic (e-)consult involve asynchronous transmission of messages between healthcare professionals to avoid a formal referral - Web-based applications  - Mobile apps | Mental health |
| Hiratsuka 2013 (95) | United States | Qualitative research | Adulthood (25-64) and Seniority (65 years+), both sexes | No | - Videoconference  - Store-and-forward | Chronic disease management |
| Hobson 2019 (20) | Australia | Systematic review | Youth (15-24), Adulthood (25-64), and Seniority (65 years +), both sexes | No | mHealth | Multiple conditions including mental health and suicide |
| Holguin 2011 (96) | United States | Non-randomised experimental study | NR | No | Store-and-forward | Traumatic brain injury |
| **Study (main author, year)** | **Country** | **Study Design** | **Participants demographics**  **(age range, sex)** | **Indigenous involvement** | **Type of technology** | **Health focus** |
| Holt 2019 (97) | Canada | Case report | Childhood (0-14 years) | Yes | Remote presence robot | Pediatric health care |
| Horn 2016 (98) | United States | Economic evaluation | NR | No | Telehealth | Telehealth to provide behavioral healthcare to rural populations |
| Humphrey 2022 (99) | New Zealand | Qualitative research | Youth (15-24), Adulthood (25-64), and Seniority (65 years +) | Yes | mHealth apps/GamblingLess | Gambling |
| Jacklin 2020 (100) | Canada | Qualitative research | Seniority (65 years +), both sexes | Yes | Remote patient monitoring (CareBand) | Dementia |
| Jacups 2017 (101) | Australia | Qualitative research | Childhood (0-14 years) | No | Videoconference and digital otoscope | Ear, nose, and throat surgery (otitis media) |
| Jacups 2018 (102) | Australia | Economic evaluation | Childhood (0-14 years) | No | - Videoconference  - Store-and-forward | Ear, nose, and throat surgery |
| Jacups 2021 (103) | Australia | Review | NR | No | Telehealth | Ear, nose, and throat diseases |
| Jaenke 2020 (104) | Australia | Mixed methods | NR | No | Mobile apps (the Store Scout app) | App to encourage healthy food choices among Australian Aboriginal and Torres Strait Islander |
| Jin 2004 (105) | Canada | Economic evaluation | All ages, both sexes | Yes | - Telephone  - Store-and-forward | Diabetes |
| Jones 2014 (106) | United States | Cross sectional study | Adulthood (25-64), women | Yes | Internet or mobile phone technology | Cardiometabolic risk after gestational diabetes |
| Jones 2017 (107) | Canada | Review | NR | No | Telehealth, mHealth, Web-based interventions, and assistive devices | NR |
| Jongbloed 2020 (108) | Canada | Cross sectional study | Adulthood (25-64), both sexes | Yes | mHealth (text-based) | Young Indigenous people who have used drugs |
| Jordan 2020 (109) | United States | Qualitative research | Adulthood (25-64) and Seniority (65 years +), both sexes | No | Store-and-forward telehealth network with a recent expansion of video telemedicine | Chronic diseases |
| Jumah 2021 (110) | Canada | Cohort study | Adulthood (25-64) | Yes | Online training program for lay maternal–infant health providers | Maternal Infant Support |
| Katapally 2020 (111) | Canada | Mixed methods | Youth (15-24), both sexes | Yes | Mobile app | Mental health |
| **Study (main author, year)** | **Country** | **Study Design** | **Participants demographics**  **(age range, sex)** | **Indigenous involvement** | **Type of technology** | **Health focus** |
| Kennedy 2021 (112) | Australia | Mixed methods | Adulthood (25-64), women | Yes | Smartphone mobile app (mHealth) | Pre pregnancy, pregnancy, and post birth |
| Kerr 2020 (113) | United States | Non-randomised experimental study | Adulthood (25-64), both sexes | Yes | Web-based training | Suicide prevention related to concerning social media posts |
| Khan 2017 (114) | Canada | Review | NR | No | Remote Presence Robotic Technology | Pediatrics, Infectious diseases (e.g., tuberculosis) |
| Kim 2015 (115) | Canada | Program report | All ages, both sexes | Yes | Store-and-forward | Diabetic retinopathy |
| Kipp 2019 (116) | Canada | Review | NR | No | - Mobile phone app: Globe Observer app, ISeeChange tracker and Local Environmental Observations - Web-based interactive maps, databases and archives - Multimedia outputs such as audio, video and photographs | NR |
| Kokesh 2011 (117) | United States | Qualitative research | NR | Yes | Store-and-forward | Ear, nose, and throat cases |
| Kruse 2016 (118) | United States | Systematic review | NR | No | - Remote monitoring - Store-and-forward - Videoconference | NR |
| Lee 2018 (119) | Australia | Qualitative research | Youth (15-24), Adulthood (25-64), and Seniority (65 years +), both sexes | Yes | Tablet app | Alcohol use disorder |
| Lee 2019 (120) | Australia | Mixed methods | Youth (15-24), Adulthood (25-64), and Seniority (65 years +), both sexes | Yes | Mobile app | Alcohol use disorder (non-drinkers, non-dependent drinkers and dependent drinkers were studied) |
| Lee 2019 (121) | Australia | Cross sectional study | Youth (15-24), Adulthood (25-64), and Seniority (65 years +), both sexes | Yes | Mobile, tablets (any app hosting device) | Alcohol use disorder |
| **Study (main author, year)** | **Country** | **Study Design** | **Participants demographics**  **(age range, sex)** | **Indigenous involvement** | **Type of technology** | **Health focus** |
| Lee 2019 (122) | Australia | Cross sectional study | Youth (15-24), Adulthood (25-64), and Seniority (65 years +), both sexes | Yes | Application on tablet device "Grog Survey App" | Alcohol use disorder |
| Legha 2020 (123) | United States | Case report | Adulthood (25-64), both sexes | No | - Videoconference - Electronic health system  - Telephone | Substance use disorder |
| Lessing 2001 (124) | Australia | Program report | Adulthood (25-64) | No | Videoconference | Psychiatry conditions unspecified |
| Levine 2009 (125) | United States | Non-randomised experimental study | Adulthood (25-64), both sexes | No | MyCareTeam diabetes management system | Diabetes |
| Lovo Grona 2004 (126) | Canada | Case report | Adulthood (25-64), women | Yes | Remote presence robot | Chronic back disorders |
| Maar 2010 (127) | Canada | Qualitative research | Adulthood (25-64), both sexes | Yes | No specific technology described | Aboriginal eHealth research agenda |
| Maar 2016 (128) | Canada | Qualitative research | Adulthood (25-64), both sexes | Yes | SMS messages | Hypertension |
| Maar 2017 (129) | Canada | Qualitative research | Adulthood (25-64), both sexes | Yes | - Bluetooth enabled blood pressure (BP) monitors - Mobile phone store-and-forward BP readings to a server - SMS messages | Blood pressure |
| Maar 2019 (130) | Canada | Randomised controlled trial | Adulthood (25-64), both sexes | Yes | SMS text messages and electronic transfer of BP measures from patients to providers | Cultural safety in electronic healthcare |
| Maar 2022 (131) | Canada | Mixed methods | Adulthood (25-64), both sexes | Yes | Simulated Cultural Communication Scenarios | Primary care |
| Macniven 2019 (132) | Australia | Mixed methods | Adulthood (25-64) | Yes | Electrocardiogram attached to a mobile phone screening device | Atrial fibrillation |
| Mah 2011 (133) | Canada | Case report | NR | Yes | Telehealth | NR |
| **Study (main author, year)** | **Country** | **Study Design** | **Participants demographics**  **(age range, sex)** | **Indigenous involvement** | **Type of technology** | **Health focus** |
| Mairs 2020 (134) | New Zealand | Qualitative research | Adulthood (25-64), both sexes | Yes | Mobile app | Behavioral problems in childhood |
| Martin 2017 (135) | Australia | Cross sectional study | Adulthood (25-64), both sexes | Yes | Online survey and phone survey | Smoking cessation |
| Mashru 2017 (136) | Canada | Program report | All ages, both sexes | No | Videoconference | Infectious diseases |
| Mathieson 2017 (137) | United States | Cross sectional study | Youth (15-24), Adulthood (25-64), and Seniority (65 years +), both sexes | No | - Retinal image scanning technologies - Mobile apps  - Activity trackers | Diabetes/diabetic retinopathy |
| McCallum 2014 (138) | Australia | Cohort study | Childhood (0-14 years), both sexes | Yes | mHealth (SMS or phone call) | Acute bronchiolitis |
| McCarthy 2010 (139) | Australia | Qualitative research | Childhood (0-14 years), both sexes | No | Videoconference | Hearing loss |
| McElfish 2019 (140) | United States | Mixed methods | Adulthood (25-64), both sexes | Yes | A video produced in Marshallese with English subtitles and disseminated through YouTube | Diabetes |
| Mealings 2020 (141) | Australia | Cross sectional study | Childhood (0-14 years), both sexes | No | Sound Scouts, a self-administered tablet-based game hearing test that screens for these deficits | Hearing loss (otitis media) |
| Mealings 2020 (142) | Australia | Non-randomised experimental study | Childhood (0-14 years), both sexes | Yes | Sound Storm iPad app | Spatial processing disorder |
| Mendez 2013 (143) | Canada | Qualitative research | NR | No | Remote presence robot | Health care access in remote settings |
| Monthuy-Blanc 2013 (144) | Canada | Qualitative research | Adulthood (25-64), both sexes | Yes | Videoconference | Mental health |
| Moo 2007 (145) | Australia | Program report | NR | No | Electronic health record | Overall health |
| Morenga 2018 (146) | New Zealand | Qualitative research | Adulthood (25-64) | Yes | Mobile app | Healthy lifestyles for Māori |
| Morenz 2019 (147) | United States | Mixed methods | NR | No | - Store-and-forward  - Videoconference | Dermatological care |
| **Study (main author, year)** | **Country** | **Study Design** | **Participants demographics**  **(age range, sex)** | **Indigenous involvement** | **Type of technology** | **Health focus** |
| Morris 2016 (148) | Canada | Qualitative research | Youth (15-24), both sexes | Yes | Social media survey | Experiences with violence (sexual, physical, and psychological abuse) |
| Moy 2010 (149) | United States | Mixed methods | Adulthood (25-64), both sexes | Yes | Remote patient monitoring | Physical activity |
| Muller 2017 (150) | United States | Randomised controlled trial | Adulthood (25-64) and Seniority (65 years +), both sexes | Yes | Text messages | Colorectal cancer |
| Muttitt 2004 (151) | Canada | Review | NR | No | No specific technology described | NR |
| Nagel 2020 (152) | Australia | Qualitative research | Adulthood (25-64), both sexes | Yes | Mobile app | Chronic kidney disease |
| Nance 2020 (153) | United States | Program report | Youth (15-24), Adulthood (25-64), and Seniority (65 years +) | No | Videoconference | Hepatitis C |
| Narva 2017 (154) | United States | Review | Youth (15-24), Adulthood (25-64), and Seniority (65 years +), both sexes | No | - Videoconference  - Zuni electronic health record | Chronic kidney disease |
| Nelson 2016 (155) | United States | Qualitative research | Age NR, women | Yes | mHealth | Obesity |
| Nghiem 2019 (156) | New Zealand | Economic evaluation | Youth (15-24), Adulthood (25-64), and Seniority (65 years +), both sexes | No | mHealth | Smoking cessation |
| Nguyen 2015 (157) | Australia | Economic evaluation | Childhood (0-14 years), Youth (15-24), and Adulthood (25-64), both sexes | No | Store-and-forward | Ear, nose, and throat |
| Nicholas 2013 (158) | Canada | Program report | NR | Yes | Videoconference | Chronic kidney disease |
| NiMhurchu 2019 (159) | New Zealand | Randomised controlled trial | Adulthood (25-64), both sexes | Yes | Mobile app | Healthy lifestyle behaviours |
| Nissen 2003 (160) | Australia | Cohort study | NR | No | Videoconference | Overall health |
| **Study (main author, year)** | **Country** | **Study Design** | **Participants demographics**  **(age range, sex)** | **Indigenous involvement** | **Type of technology** | **Health focus** |
| Noronha 2022 (161) | Canada | Scoping review | NR | Yes | Indigenous mental health mobile apps | Mental health |
| O'Halloran 2018 (162) | Australia | Retrospective observational study | Youth (15-24), Adulthood (25-64), and Seniority (65 years +), both sexes | No | Store-and-forward | Diabetic retinopathy |
| Oetzel 2019 (163) | New Zealand | Cross sectional study | Youth (15-24), both sexes | No | Mobile phone (text-based intervention) | Rheumatic fever |
| Orr 2019 (164) | United States | Randomised controlled trial | Adulthood (25-64), both sexes | Yes | mHealth (text-messaging) | Smoking cessation |
| Park 2020 (165) | Canada | Case report | Adulthood (25-64), men | No | Videoconference | Forearm emergency decompressive fasciotomy for compartment syndrome |
| Patten 2010 (166) | United States | Case report | Adulthood (25-64), women | Yes | Video and telephone | Smoking while pregnant |
| Peiris 2019 (167) | Australia | Mixed Methods | Youth (15-24), Adulthood (25-64), and Seniority (65 years +), both sexes | Yes | iPad app for screening assessments | Chronic diseases |
| Peiris 2019 (168) | Australia | Randomised controlled trial | Youth (15-24), Adulthood (25-64), and Seniority (65 years +), both sexes | Yes | Mobile app and text-message | Smoking cessation |
| Perdacher 2022 (169) | Australia | Cross sectional study | Adulthood (25-64), both sexes | Yes | Online application (Stay Strong app) via tablet | Mental health |
| Perry 2022 (170) | New Zealand | Qualitative research | Adulthood (25-64), both sexes | Yes | iSelf-help app | Pain management |
| Person 2014 (171) | United States | Program report | All ages, both sexes | No | - Store-and-forward  - Videoconference  - Telephone | Multiple conditions including otolaryngology/ head and neck surgery, urology, orthopedics, cardiothoracic surgery, neurosurgery, general, oncologic, pediatric, plastic, and vascular surgery, obstetrics, and gynecology |
| **Study (main author, year)** | **Country** | **Study Design** | **Participants demographics**  **(age range, sex)** | **Indigenous involvement** | **Type of technology** | **Health focus** |
| Phillips 2014 (172) | Australia | Randomised controlled trial | Childhood (0-14 years), both sexes | Yes | Mobile phone multimedia (videos) and text messages | Otitis media |
| Povey 2016 (173) | Australia | Qualitative research | Adulthood (25-64), both sexes | Yes | Mobile app (AIMhi Stay Strong and ibobbly suicide prevention app) | Mental health |
| Povey 2020 (174) | Australia | Mixed methods | Youth (15-24), both sexes | Yes | Mobile apps | Mental health |
| Povey 2022 (175) | Australia | Qualitative research | Childhood (0-14 years) and Youth (15-24), both sexes | Yes | Aboriginal and Islander Mental Health Initiative for Youth (AIMhi-Y app) | Mental health |
| Pruthi 2013 (176) | United States | Cross sectional study | Adulthood (25-64), women | No | - Videoconference  - Electronic health record | Breast cancer |
| Puszka 2016 (177) | Australia | Qualitative research | NR | Yes | e-Mental health | Mental health |
| Railey 2022 (178) | United States | Randomised controlled trial | Adulthood (25-64), both sexes | Yes | Home blood pressure monitoring | Hypertension |
| Raphiphatthana 2020 (179) | Australia | Mixed methods | NR | Yes | - Email  - Telephone  - Stay Strong app | Mental health |
| Raphiphatthana 2020 (180) | Australia | Qualitative research | NR | No | Authors stated: electronic mental health approaches typically involve telephone, mobile, computer, and Web-based apps | Mental health |
| Reilly 2020 (21) | Australia | Scoping review | NR | No | Web-based therapeutic interventions | Multiple conditions including cardiac care, diabetes, nutrition, mental health, smoking cessation, substance misuse, asthma, neonatal, otitis media |
| Rempel 2016 (181) | Canada | Qualitative research | Youth (15-24), women | Yes | - Telehealth nursing line  - Social media,  - Websites | First Nations young adult mothers |
| Rice 2016 (182) | Australia | Review | Youth (15-24), both sexes | No | Social media | Overall health |
| **Study (main author, year)** | **Country** | **Study Design** | **Participants demographics**  **(age range, sex)** | **Indigenous involvement** | **Type of technology** | **Health focus** |
| Roberts 2021 (183) | Canada | Qualitative research | Adulthood (25-64), both sexes | No | Telemental health services | Mental health |
| Robertson 2007 (184) | United States | Non-randomised experimental study | Adulthood (25-64), both sexes | Yes | Websites | Diabetes |
| Robinson 2011 (185) | United States | Non-randomised experimental study | Adulthood (25-64) | No | - Email  - Remote patient monitoring  - Web-based diabetes management system called MyCareTeam | Diabetes |
| Robinson 2015 (186) | United States | Non-randomised experimental study | Adulthood (25-64), both sexes | No | This paper examined patient access to health communication technologies | Overall health |
| Robler 2020 (187) | United States | Randomised controlled trial | Childhood (0-14 years) and Youth (15-24), both sexes | Yes | mHealth | Hearing loss |
| Rose 2007 (188) | United States | Cross sectional study | NR | No | - Store-and-forward  - Electronic health record | Overall health |
| Ruiz-Cosignani 2022 (189) | New Zealand | Scoping review | NR | No | - Telephone  - Videoconference  - Web-based | Mental health and or drug addiction |
| Rushing 2011 (190) | United States | Qualitative research | Youth (15-24), both sexes | Yes | Media technologies | Overall health |
| Russell 2019 (191) | Australia | Cross sectional study | Adulthood (25-64) and Seniority (65 years +), both sexes | No | Videoconference | Mental health (dementia) |
| Santiago-Torres 2022 (192) | United States | Randomised controlled trial | Youth (15-24), Adulthood (25-64), and Seniority (65 years +), both sexes | No | Smartphone apps | Smoking cessation |
| Sauve 2022 (193) | Canada | Cohort study | Adulthood (25-64) | Yes | Stand Up for Indigenous Health (SU4IH) - Mobile app | Education about social determinants of health faced by Indigenous Peoples in Canada |
| **Study (main author, year)** | **Country** | **Study Design** | **Participants demographics**  **(age range, sex)** | **Indigenous involvement** | **Type of technology** | **Health focus** |
| Savin 2006 (194) | United States | Case report | Childhood (0-14 years), both sexes | No | Videoconference | Mental health |
| Sawchuk 2008 (195) | United States | Randomised controlled trial | Adulthood (25-64), both sexes | Yes | Pedometer + telephone calls | Physical activity |
| Schaefer 2021 (196) | United States | Cohort study | Adulthood (25-64), both sexes | Yes | Home blood pressure monitoring | Hypertension |
| Sequist 2011 (197) | United States | Program report | NR | Yes | Health information technology | Multiple conditions including diabetes, alcohol-induced diseases, cardiovascular disease, cancer, communicable diseases, etc. |
| Shang 2021 (198) | Canada | Qualitative research | NR | No | Videoconference | Mental health |
| Shibasaki 2016 (199) | Australia | Program report | NR | No | Social media | Smoking prevention and cessation |
| Shore 2004 (200) | United States | Case report | Adulthood (25-64), men | No | Videoconference | PTSD, chronic alcohol dependence, with physiological dependence, sustained partial remission bereavement |
| Shore 2004 (201) | United States | Program report | Adulthood (25-64), men | Yes | - Videoconference  - Electronic health record | Mental health (PTSD) |
| Shore 2005 (202) | United States | Program report | Age NR, men | Yes | Videoconference | Mental health (PTSD) |
| Shore 2007 (203) | United States | Economic evaluation | Adulthood (25-64) and Seniority (65 years +), men | Yes | Videoconference | Mental health (psychiatric disorders) |
| Shore 2007 (204) | United States | Cohort study | Adulthood (25-64), men | Yes | Videoconference | Mental health (psychiatric disorders) |
| Shore 2008 (205) | United States | Cross sectional study | Adulthood (25-64) and Seniority (65 years +), men | Yes | Videoconference | Mental health (psychiatric disorders) |
| Shore 2012 (206) | United States | Retrospective observational study | Adulthood (25-64) and Seniority (65 years +), men | Yes | Videoconference | Mental health (PTSD) |
| **Study (main author, year)** | **Country** | **Study Design** | **Participants demographics**  **(age range, sex)** | **Indigenous involvement** | **Type of technology** | **Health focus** |
| Shore 2012 (207) | United States | Program report | Adulthood (25-64), men | Yes | - Videoconference  - Electronic health record | Mental health (PTSD) |
| Shore 2019 (208) | United States | Review | NR | No | - Videoconference  - Telephone  - Electronic health record | Mental health |
| Sicotte 2011 (209) | Canada | Cohort study | Adulthood (25-64), both sexes | No | Telehaemodialysis service:  - Videoconference  - Electronic health record | Chronic kidney disease |
| Silva 2015 (210) | United States | Cross sectional study | Adulthood (25-64), both sexes | No | Store-and-forward | Diabetic retinopathy |
| Ski 2015 (211) | Australia | Cohort study | Youth (15-24), Adulthood (25-64), and Seniority (65 years +), both sexes | No | - Telephone  - Web-based software | - Coronary heart disease  - Diabetes |
| Smith 2006 (212) | Australia | Case report | Childhood (0-14 years), both sexes | No | Store-and-forward | Ear, nose, and throat conditions |
| Smith 2012 (213) | Australia | Review | Childhood (0-14 years), both sexes | Yes | - Video-otoscope  - Wireless telecommunications | Otitis media |
| Smith 2015 (214) | Australia | Cohort study | Childhood (0-14 years), both sexes | Yes | - Video otoscope  - Secure online database | Otitis media |
| Smith 2021 (215) | Australia | Program report | NR | Yes | - Videoconference  - Educational videos | Dementia |
| Snoswell 2019 (216) | Australia | Economic evaluation | NR | No | Videoconference | Diabetes |
| Spurling 2021 (217) | Australia | Mixed methods | Childhood (0-14 years), both sexes | No | HearScreenTM (HearX, Camden, DE, USA) mobile app | Hearing loss |
| Stavrakis 2021 (218) | United States | Cohort study | Adulthood (25-64) and Seniority (65 years +), both sexes | No | Mobile single-lead ECG device | Atrial fibrillation |
| St Clair 2018 (219) | Australia | Program report | NR | Yes | Videoconference | Overall health |
| St Clair 2019 (220) | Australia | Mixed methods | NR | No | Telecommunications and telehealth services | Overall health |
| **Study (main author, year)** | **Country** | **Study Design** | **Participants demographics**  **(age range, sex)** | **Indigenous involvement** | **Type of technology** | **Health focus** |
| Stephens 2019 (221) | United States | Program report | Adulthood (25-64) | Yes | - Email  - Text messages - TeleECHO | Hepatitis C |
| Stephens 2020 (222) | United States | Randomised controlled trial | Youth (15-24), both sexes | Yes | - Text message  - Videos  - Social media | Mental health (suicide prevention) |
| Stotz 2020 (223) | United States | Qualitative research | Adulthood (25-64), both sexes | Yes | Online diabetes nutrition education platform | Diabetes |
| Sushames 2015 (224) | Australia | Cohort study | Adulthood (25-64) | No | Accelerometer | Physical activity |
| Taualii 2009 (225) | United States | Qualitative research | Youth (15-24), both sexes | Yes | SmokingZine, a web-based smoking cessation program | Cigarette smoking |
| Tighe 2017 (226) | Australia | Randomised controlled trial | Youth (15-24) and Adulthood (25-64), both sexes | Yes | Mobile app (ibboby) | Mental health (suicide prevention) |
| Tighe 2020 (227) | Australia | Qualitative research | Adulthood (25-64), both sexes | Yes | Mobile app (ibboby) | Mental health (suicide prevention) |
| Tobe 2019 (228) | Canada | Randomised controlled trial | Adulthood (25-64), both sexes | Yes | - SMS  - Blood Pressure Monitor | Hypertension |
| Tomayko 2017 (229) | United States | Randomised controlled trial | Childhood (0-14 years) | Yes | - Social media  - Text messaging  - Pedometers  - Apple corers  - Exercise DVDs | Obesity |
| Tomayko 2021 (230) | United States | Randomised controlled trial | Youth (15-24), Adulthood (25-64), and Seniority (65 years +), both sexes | No | - Text messaging  - Social media  - DVD | Obesity |
| Toombs 2021 (231) | Canada | Systematic review | Youth (15-24), both sexes | No | - Mobile app  - SMS texting  - Web-based resources  - Teleconference services | Mental health, smoking, young Aboriginal fathers, HIV, alcoholism |
| **Study (main author, year)** | **Country** | **Study Design** | **Participants demographics**  **(age range, sex)** | **Indigenous involvement** | **Type of technology** | **Health focus** |
| Tonkin 2017 (232) | Australia | Qualitative research | Youth (15-24) and Adulthood (25-64), both sexes | Yes | Mobile app | Health behavior change |
| Trees 2015 (233) | Australia | Qualitative research | Youth (15-24), both sexes | Yes | - Television advertisements  - Radio commercials | Awareness attitudes towards alcohol consumption |
| Umaefulam 2020 (234) | Canada | Mixed methods | Youth (15-24), Adulthood (25-64), and Seniority (65 years +), women | Yes | mHealth (text-message delivery platform) | Diabetes/Diabetic retinopathy |
| Umaefulam 2022 (235) | Canada | Qualitative research | Youth (15-24), Adulthood (25-64), and Seniority (65 years +), women | Yes | Text messages | Diabetes |
| Venter 2012 (236) | New Zealand | Mixed methods | NR | No | Remote patient monitoring | Congestive heart failure and chronic obstructive pulmonary disease |
| Verbiest 2019 (237) | New Zealand | Qualitative research | NR | Yes | Smartphone app | Obesity |
| Vigil-Hayes 2019 (238) | United States | Qualitative research | Youth (15-24), both sexes | Yes | ARORA (Amplifying Resilience Over Restricted Internet Access) mobile app | Mental health |
| Vigil-Hayes 2021 (239) | United States | Qualitative research | Youth (15-24), both sexes | Yes | ARORA mobile app | Mental health |
| Volpe 2014 (240) | Canada | Qualitative research | Adulthood (25-64) | No | Videoconference | Mental health |
| Weatherall 2022 (241) | Australia | Cohort study | Youth (15-24), Adulthood (25-64), and Seniority (65 years +), both sexes | Yes | Grog Survey Mobile app | Alcoholism |
| Wickramasinghe 2016 (242) | Australia | Systematic review | Seniority (65 years and above) | No | - Store-and-forward  - Computerized information kiosks  - Videoconference | Diabetes |
| Wikaire 2022 (243) | New Zealand | Qualitative research | NR | Yes | Videoconference | NR |
| Wright 2020 (244) | United States | Cross sectional study | Adulthood (25-64), both sexes | No | Geofencing SMS | NR |
| **Study (main author, year)** | **Country** | **Study Design** | **Participants demographics**  **(age range, sex)** | **Indigenous involvement** | **Type of technology** | **Health focus** |
| Wright 2021 (245) | Canada | Qualitative research | Youth (15-24), Adulthood (25-64), and Seniority (65 years +), women | Yes | Web- and App-Based Information Sources | Healthy parenting and infant health |
| Wrobel 2022 (246) | United States | Cross sectional study | Youth (15-24), both sexes | Yes | Text messages | Mental health |
| Yao 2018 (247) | United States | Mixed methods | Youth (15-24), both sexes | Yes | SMS delivery platform (Mozeo) | Sexually transmitted infections/ sexual health |
| Yazdanshenas 2016 (248) | United States | Qualitative research | Adulthood (25-64) and Seniority (65 years +), both sexes | No | mHealth (SMS) | Hypertension |
| Yilmaz 2019 (249) | United States | Economic evaluation | NR | No | Telepsychiatry | Mental health |
| Young 2016 (250) | Canada | Cohort study | Childhood (0-14 years) and Youth (15-24), both sexes | No | Customized survey app on a computer tablet | Aboriginal children’s health and well-being |
| Zheng 2022 (251) | Australia | Cross sectional study | Youth (15-24), Adulthood (25-64), and Seniority (65 years +), both sexes | Yes | Grog Survey App | Alcoholism |

**Captions.** App, Application; BP, Blood Pressure; CD-ROM, Compact Disc Read Only Memory; DVD, Digital Video Disc; ECG, electrocardiogram; e-Health, Electronic Health; e-MH, Electronic Mental Health; HIV, human immunodeficiency virus; iPad, Interactive Personal Application Device; m-Health, Mobile Health; NR, Not Reported; PTSD, Post-Traumatic Stress Disorder; SMS, Short Message Service.
